# Case Report: Combined posterior and anterior compartment separation in giant incisional hernia repair: balancing feasibility and postoperative complications

**DOI:** 10.3389/fsurg.2025.1709938

**Published:** 2025-12-18

**Authors:** Prevezanos Dionysios, Konstantinos S. Giannakopoulos, Dimitrios K. Vlachos, Georgia Marina Tsiolaki, Stylianos Kykalos

**Affiliations:** Faculty of Medicine, National and Kapodistrian University of Athens, Athens, Greece

**Keywords:** giant complex incisional hernia, Transverse Abdominis Release (TAR), anterior component separation (ACS), Botulinum toxin A, abdominal wall reconstruction (AWR)

## Abstract

**Introduction:**

The surgical management of giant incisional hernias remains a challenge, particularly in cases involving extensive defects and altered abdominal wall anatomy. The Transverse Abdominis Release (TAR) technique, particularly the Madrid modification, has emerged as a preferred approach for posterior compartment separation, allowing for wide medial mobilization while preserving neurovascular integrity. While anterior component separation (ACS) can further facilitate closure, it is associated with significant postoperative complications, including bulging, herniation, wound dehiscence, and core instability, making its use controversial. This case underscores the importance of proper pre-/post-operative management and surgical technique to avoid further complications.

**Case report:**

A 74-year-old male with a history of ruptured abdominal aortic aneurysm repair presented with a giant midline incisional hernia, with a defect measuring 17.5 cm in width, containing the left lobe of the liver and intestinal loops. Preoperative botulinum toxin injections were administered under ultrasonographic guidance, resulting in a 2.5 cm reduction in the fascial defect. A posterior approach was prioritized, utilizing Transverse Abdominis Release (TAR) with PTFE mesh reinforcement. Due to the persistent tension on the anterior sheath, limited anterior compartment separation was performed, but only to the extent necessary, given its association with high morbidity.

**Results:**

The patient had an uneventful postoperative recovery, with drain removal on day five and discharge on day eight. A minor seroma at the right costal margin was successfully managed with aspiration. Importantly, by prioritizing posterior compartment separation over extensive anterior release, we minimized the risks of wound-related complications. At 12-month follow-up, no recurrence or major complications were observed.

**Conclusions:**

This case highlights the superior role of posterior compartment separation (TAR) in achieving durable and tension-free closure of giant incisional hernias, particularly in complex cases following major vascular surgeries. Although anterior component separation remains an option, its routine use should be reconsidered due to the increased risk of complications. This case reinforces the necessity of a tailored, multidisciplinary approach, emphasizing posterior reinforcement over anterior techniques to optimize patient outcomes.

## Introduction

The surgical management of giant incisional hernias presents a unique set of challenges due to the size and complexity of the defect, often requiring innovative and tailored approaches. The Rives-Stoppa repair, introduced as a method for retro-rectus hernia repair, marked a significant advancement in the field by providing a tension-free closure with reinforcement using prosthetic mesh (1, 2). Building on this foundation, the Transverse Abdominis Release (TAR) technique—particularly the Madrid modification—has further refined the approach to large and complex hernias by allowing a wide medial mobilization of the abdominal wall with preservation of the neurovascular bundles (3). This technique enables closure of massive defects while maintaining core stability and minimizing complications.

In assessing the feasibility of hernia repair, the application of Carbonell's rule (2 × RW:DW) serves as a crucial tool for evaluating the suitability of complex repairs, especially in cases involving giant hernias (4). The rule emphasizes the need for adequate medialization to achieve successful defect closure without undue tension, thus guiding preoperative planning and technique selection.

Preoperative preparation with botulinum toxin injection has emerged as a valuable adjunct in giant hernia repair, acting as a temporary flaccid muscle paralysis without systemic effects (5). By inducing temporary paralysis of the lateral abdominal wall musculature, botulinum toxin facilitates medialization of the rectus muscles, effectively reducing the fascial defect size and enhancing the likelihood of tension-free closure. This approach has been shown to decrease the fascial defect up to 5 cm (5–7).

Giant hernias following abdominal aortic aneurysm repair represent an additional layer of complexity due to the altered anatomy, significant scar tissue, and the potential for compromised vascular integrity. In such cases, a multidisciplinary approach, including preoperative optimization with techniques such as botulinum toxin administration, becomes essential to facilitate the repair and improve outcomes.

## Case report

A 74-year-old male presented to our department with a giant incisional hernia of the linea alba. His medical history was notable for a ruptured abdominal aortic aneurysm repair performed five months prior. Additional comorbidities included atrial fibrillation, hypertension, hyperuricemia, dyslipidaemia, and depressive disorder.

**Figure 1:**
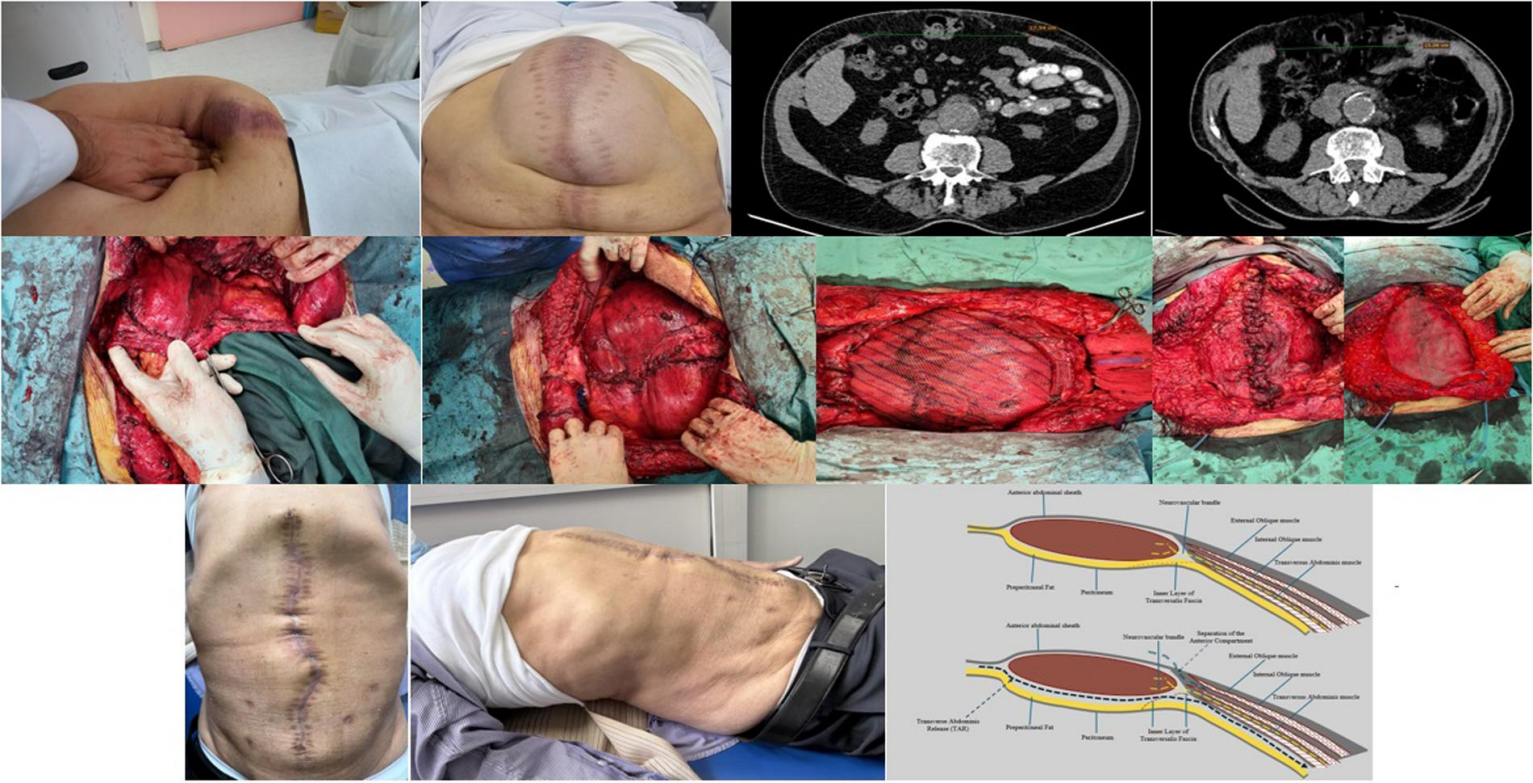
Overview of the clinical and surgical course, including preoperative assessment, intraoperative technique with mesh fixation, and a schematic representation of the repair.

On physical examination, a massive midline hernia was observed, extending from the xiphoid process to the pubic symphysis (Figure 1). Given the hernia's considerable size, a computed tomography (CT) scan was performed, revealing a midline aponeurotic ventral defect measuring up to 17.5 cm horizontally (Figure 1) (M1-M5 based on EHS classification) (8), containing the left lobe of the liver and multiple intestinal loops.

Based on the application of Carbonell's rule (RWL + RWR: DW ≥ 2:1), the patient was determined to be an appropriate candidate for Transverse Abdominis Release (TAR). To facilitate the repair and reduce the hernia defect, the patient underwent preoperative administration of botulinum toxin under Ultrasonography guidance. A 100 mg dose of botulinum toxin was injected at three different sites per side, targeting all three muscle layers—the external oblique, internal oblique, and transversus abdominis. A CT scan was performed to confirm proper needle placement (Figure 1). A repeat CT scan performed four weeks later demonstrated a reduction in the fascial defect by 2.5 cm (Figure 1).

The patient underwent a Transverse Abdominis Release (TAR) using the Madrid modification technique (3). The posterior rectus sheath was closed with PDS 2-0 sutures, and a PTFE mesh was placed to reinforce the posterior sheath, extending from the xiphoid process to the pubic symphysis (Figure 1).

Despite the success of the posterior closure, the size of the defect necessitated anterior compartment separation with extensive lateral incisions beyond the rectus abdominis muscle, detaching the external oblique muscle (Figure 1). This approach was employed while carefully considering the potential risk of core instability and related complications such as seroma formation, bulging, or lateral herniation. These risks were mitigated in this case by reinforcing the lateral incisions with an additional Pro-Grip mesh (Figure 1). The total operative time was 183 min.

Intra-abdominal pressure was monitored via bladder measurement in the recovery room and on postoperative day one, with values remaining within the normal range, confirming adequate abdominal wall compliance after reconstruction.

The decision to combine posterior and anterior compartment separation techniques underscores the complexity of the repair and highlights the necessity of tailoring the surgical approach to the unique challenges posed by giant hernias. Figure 1. Demonstrates our surgical approach.

## Results

The patient had an uneventful postoperative recovery. Redon drains were removed on the 5th postoperative day, and the patient was discharged on the 8th postoperative day in stable condition.

During follow-up, the patient developed a seroma at the right costal margin, which was successfully aspirated under ultrasound guidance. A postoperative CT scan was performed 4 weeks after surgery, confirming intact reconstruction without evidence of recurrence or other complications. The follow-up period extended for 12 months, during which the patient demonstrated favourable clinical outcomes without any other significant complications.

## Discussion

This case report describes the complexity of a postoperative hernia repair after an aortic aneurysm repair, recruiting several modalities. It has been reported that the incidence ranges from 2% to 20% and especially after aortic aneurysm repair reaches 37% (9, 10). Patients with aneurysms are known to have potential defects in collagen and elastin production, suggesting that connective tissue abnormalities may affect wound healing, predisposing patients to incisional hernia following abdominal aortic aneurysm repair (11, 12). In addition, several authors reported that the comorbidities of the patients having an abdominal aortic aneurysm repair seem to play a significant role in impaired wound healing and in the formation of the post-operative giant hernias, such as diabetes (13).

In complex giant hernias, pre-operative Botulinum toxin A (BTA) has gained popularity over the last decades. BTA induces temporary flaccid paralysis of the lateral abdominal wall muscles, leading to a significant increase in muscle length and a decrease in muscle tension. Studies have shown that this elongation effect, reaching up to 5 cm, can be crucial in reducing lateral traction forces, which are known to hinder proper wound closure and healing (7). In contrast with our case, the defect was reduced by 2.7 cm. By optimizing abdominal wall compliance, BTA reduces the need for extensive surgical techniques like component separation, thereby minimizing potential complications associated with more invasive methods (6). Furthermore, BTA has been found to maintain the integrity of the repair site during the early postoperative phase, potentially lowering recurrence rates. These outcomes make BTA an effective preoperative adjunct for improving the success and safety of hernia repair procedures (6, 7).

Combining anterior and posterior component separation (ACS and PCS) techniques is crucial for reconstructing massive and complex abdominal wall defects where midline closure is not achievable using conventional approaches. Each method serves a specific role: ACS, pioneered by Ramirez, involves the release of the external oblique aponeurosis to advance the anterior rectus sheath, while PCS, including transversus abdominis release (TAR), aims to increase medialization of the posterior rectus sheath by separating deeper muscle layers.

A key study using post-mortem human specimens demonstrated that combined component separation achieved a cumulative median medialization of 5.8–9.2 cm for the anterior rectus sheath and 10.1–14.2 cm for the posterior rectus sheath, depending on the abdominal level. In cases where ACS was performed after PCS, an additional 32% to 38% of anterior sheath advancement was achieved (14). Conversely, PCS after ACS yielded a further 50% to 59% medialization of the posterior rectus sheath (14). Despite these anatomical gains, studies emphasize that the incremental benefits of combining both techniques must be weighed against additional surgical risks, such as increased wound morbidity and destruction of tissue planes.

Clinical data from a retrospective analysis of 12 patients undergoing both ACS and PCS for complex hernia repair revealed no hernia recurrence over a mean follow-up of 27 months. This retrospective study, with a mean hernia width of 23.5 cm, experienced a postoperative surgical site infection (SSI) rate of 33.3%, a seroma incidence of 58.3%, and wound dehiscence in 25% of cases. Despite these complications, patients reported statistically significant improvements in quality of life scores for pain, restrictions and cosmetic appearance (15).

These findings underscore the potential of combining ACS and PCS to achieve optimal tension-free closure in extreme cases but also highlight the need for rigorous preoperative preparation and multidisciplinary management to mitigate complications.

## Conclusion

The tension-free Transverse Abdominis Release (TAR) technique played a pivotal role in achieving posterior compartment closure, providing robust structural support and reducing the risk of recurrence. The combination of TAR with anterior compartment separation, facilitated by extensive lateral incisions and additional mesh reinforcement, allowed for effective reconstruction of both the posterior and anterior abdominal wall, addressing the limitations of primary closure in such a massive defect.

This case highlights the importance of a tailored, multidisciplinary approach in addressing complex hernia repairs. The successful outcome achieved, with minimal complications, emphasizes the utility of advanced surgical techniques and careful perioperative management in overcoming the significant challenges posed by giant hernias.

## Data Availability

The original contributions presented in the study are included in the article/Supplementary Material, further inquiries can be directed to the corresponding author.
